# Low-level contamination confounds population genomic analysis

**DOI:** 10.1093/g3journal/jkag021

**Published:** 2026-01-30

**Authors:** Audrey K Ward, Eduardo F C Scopel, Brent Shuman, Michelle Momany, Douda Bensasson

**Affiliations:** Department of Genetics, University of Georgia, 120 E. Green St., Athens, GA 30602, United States; Institute of Bioinformatics, University of Georgia, 120 E. Green St., Athens, GA 30602, United States; Department of Plant Biology, University of Georgia, 2502 Miller Plant Sciences, Athens, GA 30602, United States; Department of Plant Biology, University of Georgia, 2502 Miller Plant Sciences, Athens, GA 30602, United States; Institute of Bioinformatics, University of Georgia, 120 E. Green St., Athens, GA 30602, United States; Department of Plant Biology, University of Georgia, 2502 Miller Plant Sciences, Athens, GA 30602, United States

**Keywords:** phylogenomic, population genomics, heterozygosity, BAF plots, population structure, single nucleotide polymorphism (SNP) calls, cross-contamination

## Abstract

Genome sequence contamination has a variety of causes and can originate from within or between species. Previous research focused on contamination between distantly related species or on prokaryotes. Here, we test for intraspecies contamination by mapping short read genome data to a reference and visualizing the frequency of reads with single nucleotide differences from the reference. Out of 1,298 publicly available genome sequences investigated for *Saccharomyces cerevisiae*, a small number (eight genomes) show at least 5% contamination. Contamination rates differed however among sequencing centers: one unusually large study had a low contamination rate (below 0.2%) but the contamination rate was higher for other studies (2% or 15% of genomes). Using genome data contaminated in silico to known degrees, we showed that contamination is recognizable in plots with unexpected secondary allele (B-allele) frequencies of at least 5% and measured contamination effects on admixture and phylogenetic analysis in two fungal species. With a standard base calling pipeline, we found that contaminated genomes superficially appeared to produce good quality genome data. Yet as little as 5–10% genome contamination was enough to change phylogenetic tree topologies and make contaminated strains appear as hybrids between lineages (genetically admixed). We recommend the use of B-allele frequency plots to screen genome resequencing data for intraspecies contamination.

## Introduction

The contamination of high-throughput sequence data is a known challenge in genome biology that can lead to incorrect inferences (Merchant et al. 2014; Wilson et al. 2018; Goig et al. 2020; Prous et al. 2020). Low level sample contamination can occur in laboratories during DNA extraction or in culture, at sequencing centers during amplification steps, or even in silico if barcodes are not easily distinguished after multiplexing (Dickins et al. 2014; Ballenghien et al. 2017; Clark et al. 2019; Cornet and Baurain 2022). Most existing tools detect contamination that occurs between species (Cornet and Baurain 2022). Yet analysis of bacterial genomes suggests within-species contamination is more likely to lead to mistakes in base calling, species identification or phylogenetic analysis (Pightling et al. 2019). Furthermore, analysis of RNAseq data for animal mtDNA shows that intraspecies contamination can result in the overestimation of heterozygosity and incorrect inference of balancing selection (Ballenghien et al. 2017).

Few tools detect intraspecies contamination, and mostly this is by comparison of read data to sequence databases for prokaryotes or particular genes or species (Cornet and Baurain 2022) and therefore cannot be applied to many organisms. A possible alternative is to identify short read data with unusual frequencies of variant alleles after mapping to a reference (Dickins et al. 2014). Visualizations of variant (or B) allele frequencies in plots showing SNPs relative to reads mapped to a reference are commonly used to determine ploidy or aneuploidy (Zhu et al. 2016; Bensasson et al. 2019). We find these can also be used to distinguish genome data with unexpected B-allele frequencies. Using B-allele frequency plots, we encountered low level intraspecies contamination in public data for two model fungal species; *Saccharomyces cerevisiae* and *Aspergillus fumigatus*.

To determine whether low levels of intraspecies contamination are cause for concern, we tested the sensitivity of a standard base calling pipeline, admixture and phylogenomic analyses to within-species contamination using read data that we contaminated in silico to known degrees (0–50%). Such mixtures could result in laboratories or genome sequencing centers through sample cross-contamination at any stage of the process needed to extract or amplify DNA for genome sequencing.

## Methods

**Fig. 1.**
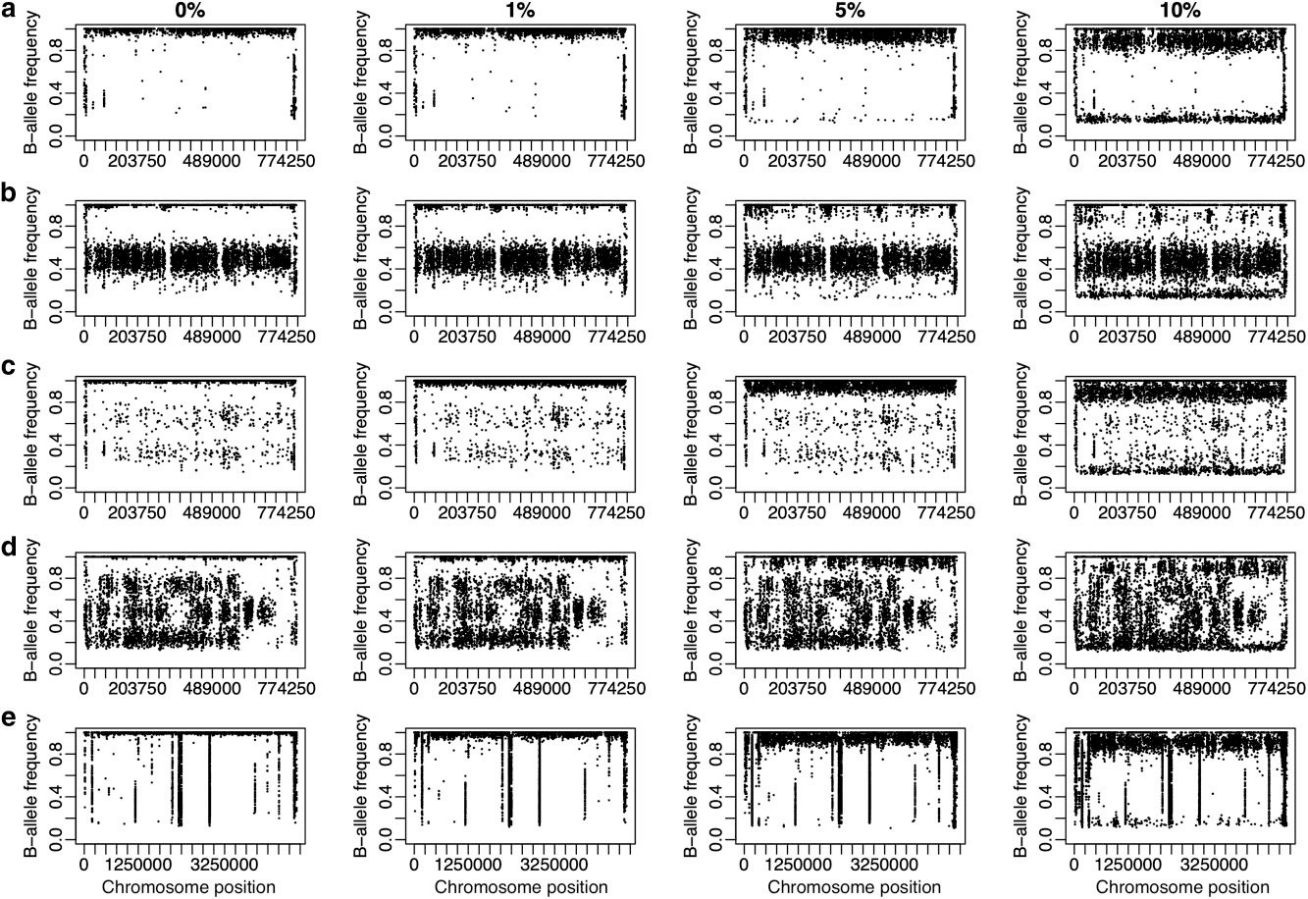
Intraspecies contamination is recognizable in B-allele frequency plots at 5% contamination. Plots show base calls for resampled genome data contaminated in silico to: 0%, 1%, 5%, and 10%. Points show the frequency of nonreference “B” alleles along the whole of chromosome II for *S. cerevisiae* a–d) and along all of chromosome 1 for *A. fumigatus* (e) for different ploidy levels: a) a haploid, b) diploid, c) triploid, d) tetraploid, and e) haploid. In contaminated mixtures, a substantial fraction of SNP differences from the reference genome appear below their expected frequency of 1.0, at the level expected if the contaminating strain has the same allele as the reference e.g. 0.95 for 5% contamination with a strain matching the reference. In repetitive regions, variants appear at many allele frequencies appearing as vertical lines on the plots.

**Fig. 2.**
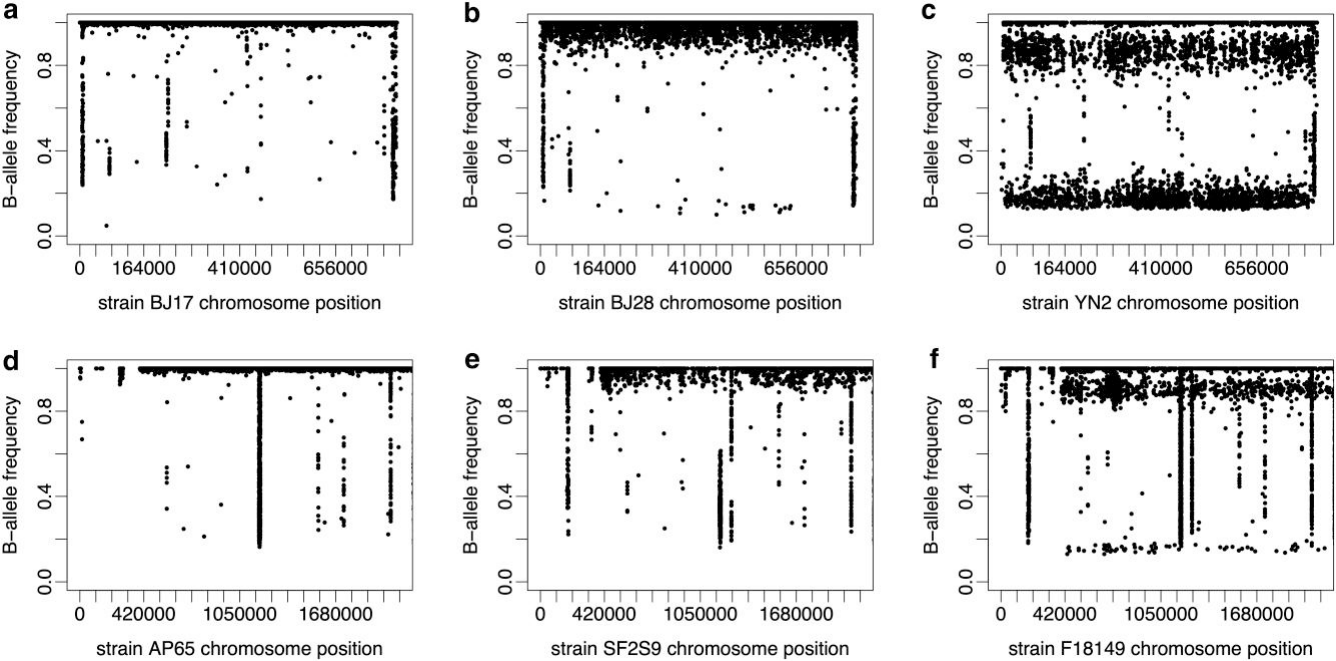
B-allele frequency plots show intraspecies contamination in public genome data. Plots show base calls for public genome data for *S. cerevisiae* chromosome II (a–c; SRA project PRJNA396809) and *A. fumigatus* chromosome 1 (d–f; SRA project PRJEB1497). Comparison with Fig. 1 suggests no contamination for strains BJ17 and AP65 (a and d); low-level contamination around 5% for strains BJ28 and SF2S9 (b and e); and at least 10% contamination for strains YN2 and F18149 (c and f). Median read depth at SNPs was 165× for strain BJ17 a), 97× for strain AP65 b), 80× for strain BJ28 c), 88× for strain YN2 d), 75× for strain SF2S9 e), and 105× for strain F18149 f).

Previously, whole genome sequences from over 1,000 *S. cerevisiae* strains and several studies (Almeida et al. 2015; Barbosa et al. 2016; Duan et al. 2018; Peter et al. 2018; Peña et al. 2025) were each mapped to a reference; SacCer_Apr2011/sacCer3 from strain S288c at UCSC as described in Peña et al. (2025). Genome data were visually inspected one by one to check for aneuploidy (Scopel et al. 2021) and intraspecies contamination (Peña et al. 2025) using vcf2alleleplot.pl (Bensasson 2018). Here, a genome sequence with an unexpected genome-wide B-allele frequency of at least 5% was defined as contaminated: see Results and discussion, Figs. 1 and 2 below, Zhu et al. (2016), or Bensasson et al. (2019) for further explanation of B-allele frequencies. We performed the experiments for Peña et al. (2025) and this study in parallel. For the Peña et al. analyses, we had access to over 1,000 genome sequences, only needed a smaller number for further analysis, and did not yet know at what threshold contamination could lead to incorrect phylogenetic or admixture results. The threshold for Peña et al. was therefore very conservative at 1%, but that was not a problem because our goal was to select a small subset of the total data. For this study, we originally tested 1% (no downstream problems) and 10% (see discussion of problems below). We then repeated our analyses at 5% and showed that at 5% there were still discernable effects on phylogenetic analyses. We do not recommend a 1% threshold because in cases where read depth is low (e.g. 30–50×) it is hard to distinguish contamination from error around 1.0. For *A. fumigatus*, we did not similarly screen large numbers of genome sequences but did encounter more contamination examples (e.g. Fig. 1d–f).

To understand the effects of intraspecies contamination on base calls and phylogenomic analysis, we created contaminated mixtures with various levels of contamination for *A. fumigatus* and *S. cerevisiae*; 0, 1, 5, 10, 20, 30, 40, and 50%. Using published short-read data (Scopel et al. 2021; Kang et al. 2022; Supplementary Table S1), *S. cerevisiae* haploid, heterozygous diploid, triploid, and tetraploid genomes (CBS1479, DBVPG1074, NPA05a1, UCD_06-645) were contaminated with reads from a haploid strain (CLIB219.2b). For *A. fumigatus*, the contaminant and recipient were haploid strains eAF749 and eAF163, respectively. The contaminating strain for each species was chosen so that it was genetically distinct from recipient strains and cross-contamination could therefore be readily recognized. The reads for each mixture were randomly sampled without replacement using seqtk sample (v1.2 for *S. cerevisiae*; 1.3 for *A. fumigatus*; https://github.com/lh3/seqtk). For each *S. cerevisiae* strain (12 Mbp genome), we used 8 million paired reads; 4 million for each simulated fastq file. For each *A. fumigatus* (29 Mbp genome), we used 6 million reads; 3 million for each simulated fastq file.

For base calling, we mapped reads to reference genomes using Burrows-Wheeler Aligner (bwa mem, v0.7.17; Li and Durbin 2009). The reference genomes were sacCer3 for *S. cerevisiae* and ASM265v1 from strain Af293 for *A. fumigatus* (Nierman et al. 2005). For SNP calling in bacteria, sometimes multiple reference genomes are needed because of their high intraspecies genetic diversity (Valiente-Mullor et al. 2021), but nucleotide diversity is low for the eukaryotes used here. For both species, the haploid recipient strains are more closely related to the reference strains (*S. cerevisiae*: 99.5% identity; *A. fumigatus*: 99.8% identity) than contaminant strains (*S. cerevisiae*: 99.3% identity; *A. fumigatus*: 99.7% identity). This means that contaminating reads are less likely to map to the reference than those of the original strain. Consensus sequences were generated using SAMtools mpileup and BCFtools call -c (v1.6; Li et al. 2009) with indels removed and read depth limited to a maximum of 100,000 reads. Mapped alignments were converted to fasta format consensus sequences using vcfutils.pl vcf2fq (from BCFtools) and seqtk seq with a phred-scaled quality threshold of 40 to define low quality base calls. Mitochondrial DNA was removed for downstream analyses. We generated BAF plots using vcf2alleleplot.pl with default options and counted high quality heterozygous and homozygous sites in fasta files using basecomp.pl (Bensasson 2018).

To test the effects of contamination on population structure, genetic admixture and phylogenetic analyses, we compared each recipient or contaminated genome to a panel of reference strains with known phylogenetic positions (Supplementary Table S1). In general, strains assigned to known clades showed unchanging phylogenetic positions when considering individual genes, larger loci, whole chromosomes or genomes in *S. cerevisiae* (Scopel et al. 2021; Peña et al. 2025) suggesting variation in gene content does not affect whole-genome tree topology in this species. For *S. cerevisiae*, we randomly selected up to two strains (where available) from each of the known 26 lineages described in Scopel et al. (2021), which resulted in a total of 52 reference panel strains including the contaminant strain. Recent genetic admixture is common in *S. cerevisiae* (Liti et al. 2009) and can complicate phylogenetic analysis, but prior analyses show that none of the strains used here were admixed (Peter et al. 2018; Scopel et al. 2021). For *A. fumigatus*, we used one-dimensional k-means clustering to categorize 168 strains (Kang et al. 2022) into 52 clusters based on their pairwise genetic distances from a single strain (CF098), then randomly chose a single strain from each cluster. Genetic distances were estimated using the dnadist function of PHYLIP (v3.697) with default parameters and a 0.5:1 transition:transversion ratio (Felsenstein 1993), and we used python to perform the k-means clustering (getGenDist.py; Scopel 2024). Because consensus genome sequences for reference panel genomes as well as contaminant and recipients were generated by mapping to reference with insertions and deletions removed (see above), all sequences were mapped to the same coordinates as the reference genomes so no separate alignment step was necessary.

For analysis of population structure and genetic admixture, we examined only the effect of contamination on the haploid *S. cerevisiae* recipient genome using ADMIXTURE (v1.3.0) (Alexander et al. 2009). Resampled (0%) or contaminated (5% and 10%) genome sequences were each merged into an alignment with the sequence of 52 reference panel strains using BCFtools view with the –min-ac 1 option (v1.15.1). Low-quality reads (phred score under 40) were filtered in VCFtools (v0.1.16; Danecek et al. 2011). Alignment files were converted to text and binary files using PLINK (v1.9b_6.21; Purcell et al. 2007). Genomes were assigned to populations (genetic clusters) in repeated ADMIXTURE runs with default parameters and varying numbers of populations (K); from 2 to 26 with five replicates per K. Resultant ancestry proportions were aligned across K values using CLUMPAK distruct (v1.1; Kopelman et al. 2015) and results were visualized using R (v4.3.3) and the R package pophelper (v2.3.1; Francis 2017).

For phylogenetic analyses, we applied neighbor-joining distance and maximum likelihood approaches to the haploid *S. cerevisiae* and *A. fumigatus* recipient genomes. Neighbor-joining trees were constructed using MEGA (v10.0.5; Kumar et al. 2016) with the Tamura-Nei model (Tamura and Nei 1993) and 100 bootstrap replicates. Maximum likelihood trees were estimated using RAxML (v8.2.11 for *S. cerevisiae* and 8.2.12 for *A. fumigatus*; Stamatakis 2014) with a GTRGAMMA model and 100 bootstrap replicates. For visualization, trees were rooted with EN14S01, GE14S01 7B and HN6 for *S. cerevisiae* and JN10 for *A. fumigatus* then right ladderized using the ape package (v5.8; Paradis and Schliep 2019) in R. The Fisher’s exact test, other analyses and visualizations were also performed in R.

## Results and discussion

### Within-species contamination in public short read genome data

B-allele frequency plots are routinely used to distinguish homozygous or haploid genome data from heterozygous diploids or polyploids (Zhu et al. 2016; Bensasson et al. 2019). In the absence of contamination, single nucleotide polymorphisms (SNPs) in homozygous diploids and haploids (Fig. 1a and e) are expected to differ from the reference genome at read allele frequencies of 1.0; in heterozygous diploids at frequencies of 1.0 or 0.5 (Fig. 1b); triploids at 1.0, 0.67, or 0.33 and so on (Fig. 1c,d). We expect that small amounts of contaminating genomic DNA from another individual will result in intermediate B-allele frequencies across the whole genome. These unexpected variants are especially clear at levels of 5% or 10% in B-allele frequency plots (Fig. 1).

In screening short-read genome data for aneuploidy, we observed public read samples with B-allele frequencies at appreciable levels (roughly 5% or more) in *S. cerevisiae* (Fig. 2b and c, Table 1) and *A. fumigatus* (Fig. 2e and f) and not at the discrete levels expected with changes in ploidy. This is likely the result of sample cross-contamination and does not improve with the trimming of low quality, unpaired reads or adapters (Supplementary Fig. S1). Outside the laboratory, *S. cerevisiae* are mostly homozygous diploids and *A. fumigatus* strains are usually haploid, and ploidy changes are rare. We screened 1,298 *S. cerevisiae* genome samples sequenced to high read depth (over 30×; Peña et al. 2025) and found 8 genomes with at least 5% intraspecies contamination. Most of these (N=6) showed 5–10% contamination, and two showed 10–20% contamination (Table 1). Higher levels of contamination would be difficult to distinguish from polyploidy using our methods but are probably less likely.

**Table 1.** Differences among studies in rates of intraspecies *Saccharomyces cerevisiae* contamination.

SRA identifier	Uncontaminated	Contaminated^a^	%	Study^b^
ERP014555	915	0	0	Peter et al. (2018)
PRJNA396809	260	5^c^	2	Duan et al. (2018)
PRJEB7601	55	0	0	Almeida et al. (2015)
PRJNA1090965	43	0	0	Peña et al. (2025)
PRJEB11698	17	3^d^	15	Barbosa et al. (2016)

^a^ Genome sequence sample had at least 5% contamination.

^b^ Data also summarized in Peña et al. (2025) after excluding genomes with low read depth and studies with fewer than 10 high depth genomes. Rates appear different (Fisher’s exact test, $P=2\times 10^{-6}$) even after excluding PRJEB11698 (Fisher’s exact test, P=0.002).

^c^ strains BJ28, CJM19.6, GS3, SX11 (5–10% contaminated), YN2 (10–20% contaminated).

^d^ strains UFMG-CM-Y645, UFMG-Y651 (5–10% contaminated), UFMG-CM-Y456 (10–20% contaminated).

To our knowledge, the extent of intraspecies contamination in public genome data has not been reported for eukaryotes. The average percentage of public *S. cerevisiae* sequence samples (0.62%; 8 out of 1,298) with at least 5% intraspecies contamination is lower than those previously reported for bacteria at levels expected to affect base calling (*Escherichia coli* for 0.87%, *Salmonella enterica* 1.48%, *Listeria monocytogenes* 2.22%; Pightling et al. 2019). The percentage of *S. cerevisiae* read samples with contamination do however vary greatly by study: from under 0.2% to 15% (Table 1; Fisher’s exact test, $P=2\times 10^{-6}$). This is consistent with past observations that the extent of contamination can differ substantially among studies and sequencing centers (Ballenghien et al. 2017; Goig et al. 2020). The percentage of contaminated genomes that is acceptable may depend on the scientific question addressed. For example, if a study is testing whether sexual reproduction is occurring in a putatively asexual species, then even a low percentage of contaminated genomes could lead to wrong conclusions.

### The effects of in silico contamination on base calling

Most contaminated data show only low levels of contamination (5–10%; 6 out of 8 contaminated genomes), so correct base calls outnumbered incorrect calls by ten to twenty fold. To determine whether such low level contamination impacts base calling, we examined in silico simulations of read data with known levels of added contamination using a standard base calling pipeline. We applied a phred-scaled quality filter (Q40) that labels sites as “low quality” data if they have estimated error rates above 1 in 10,000; a consensus base call would be represented with an “N” and therefore treated as missing data in downstream analyses. The proportion of low quality base calls does not increase with increasing levels of contamination (Supplementary Table S2). The number of high quality heterozygous base calls does increase with increasing contamination, but in haploids and triploids heterozygosity only reaches the levels seen in diploids and tetraploids with 20% contamination or above. Surprisingly, even the number of high quality homozygous base calls increases slightly at 20% contamination for haploids, and with any amount of contamination at higher ploidy levels (Supplementary Table S2). These simple quality checks that are easily performed without population genomic analyses suggest that contamination at the levels usually observed in public databases do not greatly affect base calling. However these checks do not address the effects of contamination on variant or SNP sites in particular, which are likely affected differently than invariant sites and are critical for downstream applications.

### Low-level contamination affects population genomic analyses

Intraspecies contamination likely results in erroneous heterozygous calls at SNP sites. It is therefore not surprising that contamination in past work led to mistakes in estimating the inbreeding statistic, ${\text{F}}_{IT}$, which relies on correct heterozygous base calls (Ballenghien et al. 2017). Other important population genomic statistics, such as Tajima’s D and ratios of nonsynonymous to synonymous diversity were less sensitive to intraspecies contamination (Ballenghien et al. 2017). The effects of contamination on other population genomic analyses have not previously been reported.

**Fig. 3.**
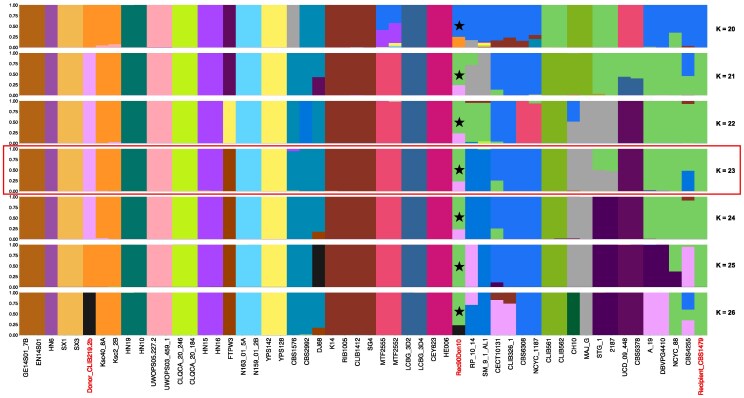
Contamination levels of 10% result in incorrect calls of genetic admixture. The plot shows the ancestry proportions for each *S. cerevisiae* individual arranged in the order seen in maximum likelihood phylogenetic analyses Supplementary Fig. S4. These plots show the runs with the highest log-likelihoods for each assumed number of populations (*K* = 20–26), and the run with the most clustering similarity to the phylogenetic analyses (*K* = 23) is highlighted with a rectangular box. Individual genomes highlighted with red text are the contaminant genome (Donor_CLIB10_2B), and the recipient genome showing the ancestry proportions expected with 0% contamination (Recipient_CBS1479) and an in silico mix of recipient with 10% contamination from the contaminant (Rec90Don10, column labelled with a star). In most runs, the genome with 10% contamination shows 25% admixture between contaminant and recipient lineages.

Here, we investigate the effects of contamination on the inference of population structure and genetic admixture from allele frequency data. Such analyses use heterozygous base calls (Pritchard et al. 2000; Alexander et al. 2009) and are therefore likely sensitive to contamination. We tested the effect of contamination at 5% and 10% contamination on the inference of individual ancestry from allele frequency data for *S. cerevisiae* using the software ADMIXTURE (Alexander et al. 2009). In all ADMIXTURE runs, 5% contamination did not affect results (Supplementary Fig. S2). The estimation of ancestry was affected however by 10% contamination. In most runs, the contaminated strain appeared admixed between contaminant and recipient lineages and mostly to a greater extent (25%) than the expected 10% contamination level (Fig. 3).

In contrast to allele frequency analyses, we expect phylogenetic analyses to be more robust to low levels of contamination because most phylogenetic software treat heterozygous sites as missing data, and we do not expect low-level contamination to result in homozygous calls for the minority allele. To test the impact of contamination on phylogenetic analysis, we included a contaminated strain in within-species phylogenomic trees for *A. fumigatus* and *S. cerevisiae*.

**Fig. 4.**
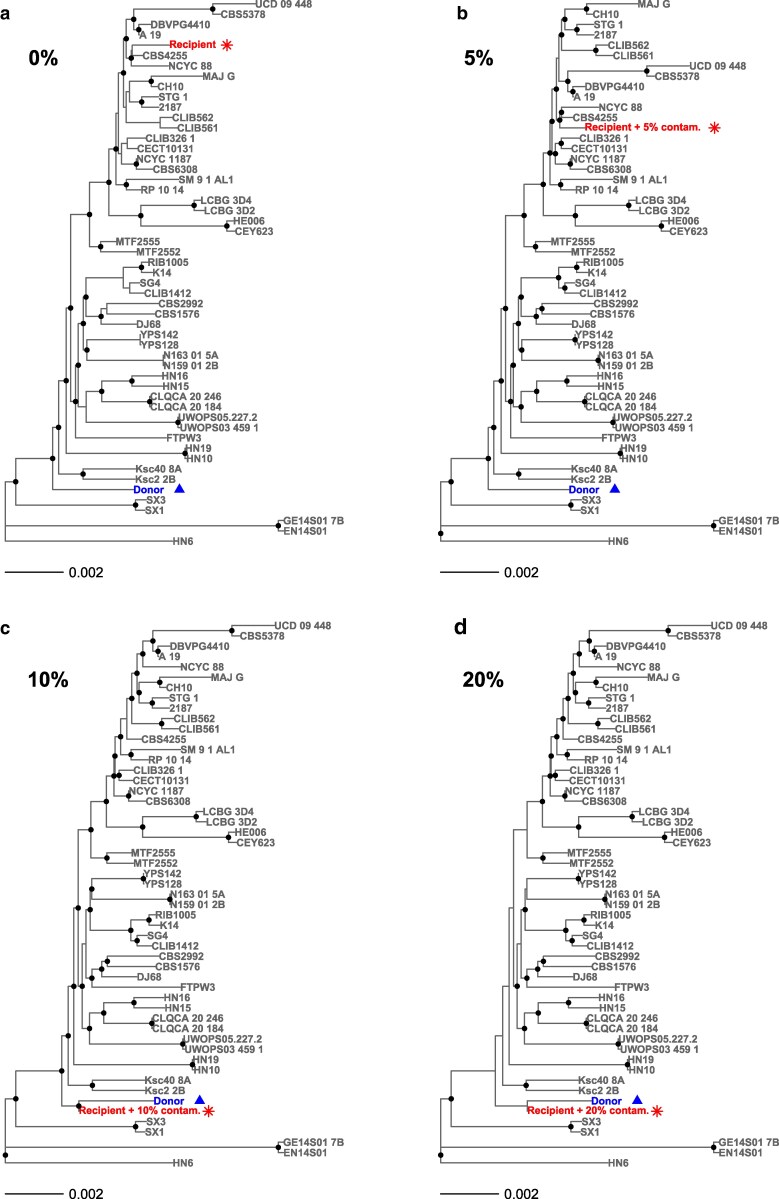
Change in topology for neighbor joining *S. cerevisiae* phylogenetic trees starting at 5% contamination. a) Contaminant and recipient genomes in the absence of contamination; b) the recipient with 5% contamination results in a tree with slightly altered topology; c) and d) at higher levels of contamination, 10% and 20%, the recipient clusters with the contaminating strain. Dots at nodes represent bootstrap support of at least 95%.

Surprisingly, the phylogenetic placement of the recipient strain changed considerably even with only 10% contamination (Fig. 4, Supplementary Figs. S3–S5). This was true for *S. cerevisiae* and *A. fumigatus* using neighbor joining distance or maximum likelihood approaches. At 10% contamination, we observed major shifts in phylogenetic position (Fig. 4, Supplementary Figs. S3–S5) and by 20% contamination the recipient *S. cerevisiae* strain clustered with the contaminating strain (Fig. 4 and Supplementary Fig. S4). For *A. fumigatus*, we did not include the contaminating strain in the phylogeny, yet we still saw major changes to tree topology (Supplementary Figs. S3 and S5). Using neighbor-joining distance, we even saw a small effect on tree topology at 5% contamination in *S. cerevisiae* (Fig. 4).

How could low-level (5–10%) contamination alter tree topology? In contaminated data, differences between the contaminant and recipient sequence appear as heterozygous sites (Supplementary Table S2; Ballenghien et al. 2017). These sites no longer contribute to estimates of genetic distance between the contaminated strain and contaminating lineage using most phylogenetic software (Lischer et al. 2014). In addition, the contaminant alleles will be called in regions where the recipient genome has low quality sequence or deletions relative to the reference, which could explain the increase in homozygous base calls at increasing levels of contamination (Supplementary Table S2). In cases where the donor genome has more high quality regions mapping to the reference than the recipient genome (as in this study; Supplementary Table S2) enough homozygous base calls might result from contaminating reads to change tree topology. The chances of seeing an effect of contamination on cluster analyses also increase with increasing divergence between contaminant and recipient genomes (Pightling et al. 2019). The strains we used in this study are from genetically distinct lineages (Fig. 4, Supplementary Figs. S2–S5), so our analyses probably represent a worst-case scenario.

## Conclusions

Here, we show that within-species contamination of genome data can lead to incorrect phylogenies or inference of genetic admixture, even at the low levels seen in public databases. Contamination has led to incorrect conclusions in the past, but most reports are on between-species contamination or Sanger sequencing studies (Merchant et al. 2014; Wilson et al. 2018; Goig et al. 2020; Prous et al. 2020). Analysis of contamination in bacteria show that within-species genome contamination can be especially damaging (Pightling et al. 2019). Using eukaryotic models, we show that intraspecies contamination can lead to the incorrect inference of genetic admixture from allele frequencies (Fig. 3) and that phylogenetic analyses can be more sensitive to contamination (5–10%, Fig. 4) than previously recognized (40–50%; Pightling et al. 2019). Contamination could affect within-species phylogenetic analysis of asexual or inbred species (e.g. bacteria, fungi, viruses), or organelle genomes (e.g. mitochondria, chloroplasts). Within-genus contamination may also lead to similar problems for phylogenetic analysis of obligately sexual species such as animals (Goig et al. 2020). Once identified, contaminated samples can be excluded from downstream analysis or it is possible to clean data (Dickins et al. 2014). A quantitative bioinformatics tool that could detect contamination in population genomic data would be ideal. For now, the visualization of SNPs in mapped read data with B-allele frequency plots provides a means to synchronously assess ploidy, heterozygosity, and potential contamination (Fig. 1); all important information for downstream phylogenetic or population genomic analyses.

## Supplementary Material

jkag021_Supplementary_Data

## Data Availability

SRA accession numbers for the data used are listed in Additional file 1: Supplementary Table S1. The code developed in this project, getGenDist.py, is available on GitHub and archived at Zenodo with doi 10.5281/zenodo.13881957 under a Creative Commons Attribution International license (CC BY 4.0). Supplemental material available at G3 online.
