# Supplementary material for: Low-level contamination confounds population genomic analysis
Source: G3 (Bethesda). 2026 Jan 30;16(4):jkag021. doi: 10.1093/g3journal/jkag021 (PMC13042315; doi:10.1093/g3journal/jkag021)
Supplement: jkag021_Supplementary_Data [file jkag021_supplementary_data.zip › Table_S2,_Figures_S1-S5_G3-2025-406484.pdf]

Ward *et al.* Supplemental Tables and Figures

Table S2: Intra-species contamination does not lower the quality of base calls for *S. cerevisiae* or *Aspergillus fumigatus*

% contaminant[1]	Ploidy	Homozygous	Heterozygous	Low Quality	P(LQ)[2]
A. fumigatus					
100%	haploid	27,651,544	22,721	1,699,323	0.058
0%	haploid	26,882,110	19,084	2,482,752	0.084
1%		26,879,682	19,161	2,485,103	0.085
5%		26,874,276	19,869	2,489,799	0.085
10%		26,874,060	22,582	2,487,309	0.085
20%		26,897,303	31,975	2,454,719	0.084
30%		26,933,648	40,079	2,410,224	0.082
S. cerevisiae					
100%	haploid	11,461,857	3,697	604,481	0.050
0%	haploid	11,430,299	2,706	638,192	0.053
1%		11,432,806	2,784	635,631	0.053
5%		11,448,751	4,868	617,607	0.051
10%		11,456,000	24,042	591,174	0.049
20%		11,464,068	72,671	534,541	0.044
30%		11,471,388	80,901	519,008	0.043
40%		11,473,538	81,923	515,830	0.043
50%		11,474,291	82,281	514,720	0.043
0%	diploid	11,426,767	62,889	581,602	0.048
1%		11,427,772	62,895	580,591	0.048
5%		11,431,346	64,035	575,878	0.048
10%		11,430,347	76,131	564,783	0.047
20%		11,435,185	104,413	531,692	0.044
30%		11,439,882	108,814	522,575	0.043
40%		11,441,299	108,547	521,427	0.043
50%		11,441,050	105,473	524,656	0.043
0%	triploid	11,421,825	8,610	637,335	0.053
1%		11,423,877	8,665	635,663	0.053
5%		11,425,285	10,582	631,913	0.052
10%		11,415,094	28,950	624,236	0.052
20%		11,413,683	75,713	579,163	0.048
30%		11,419,975	83,775	564,165	0.047
40%		11,423,533	84,218	559,800	0.046
50%		11,423,917	83,808	560,476	0.046
0%	tetraploid	11,347,790	46,526	676,765	0.056
1%		11,349,837	46,590	674,801	0.056
5%		11,376,595	47,656	646,986	0.054
10%		11,414,485	59,758	596,994	0.049
20%		11,439,265	96,163	535,856	0.044
30%		11,446,409	101,228	523,665	0.043
40%		11,449,966	98,988	522,346	0.043
50%		11,452,171	95,042	524,088	0.043

[1] Percent of reads from the contaminant (donor) genome. The recipient genome has 0% of reads from the contaminant.
[2] Proportion of base calls that are low quality (phred-scaled quality lower than Q40).

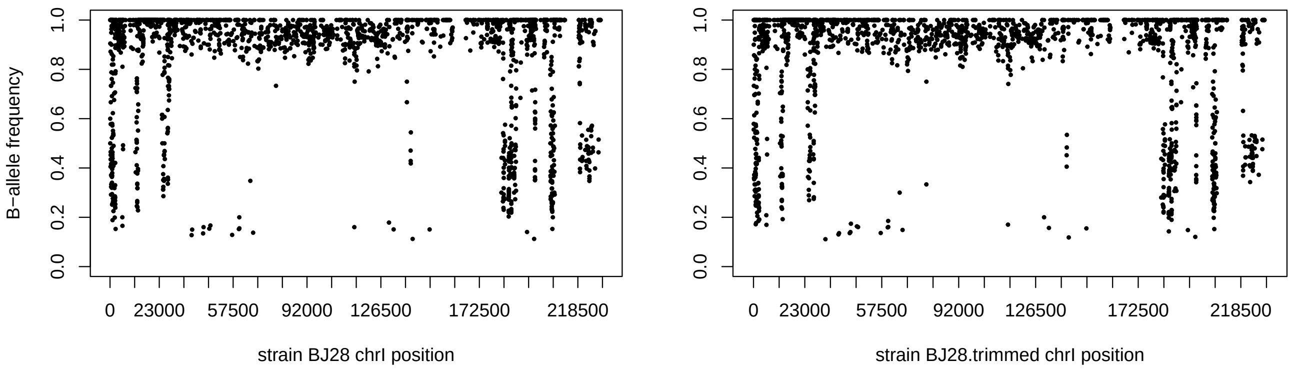

Figure S1: **No effect of read trimming on contamination levels.** Plots show base calls for public genome data for *S. cerevisiae* strain BJ28 chromosome II (SRR7851690) . Both plots show base calls at 1.0 and roughly around 0.95 suggesting low-level (ca. 5%) contamination. For the left hand plot, reads were mapped directly to the reference without first trimming reads. The right hand plot shows base calls inferred from reads that were mapped to the reference after removing unpaired reads, and trimming to remove low quality bases and adapter sequences. There is no clear difference between the two plots suggesting that cross-contamination is not the result of low quality reads. Reads were trimmed using Trimmomatic version 0.39-Java-17 with default parameters (Bolger *et al.*, 2014).

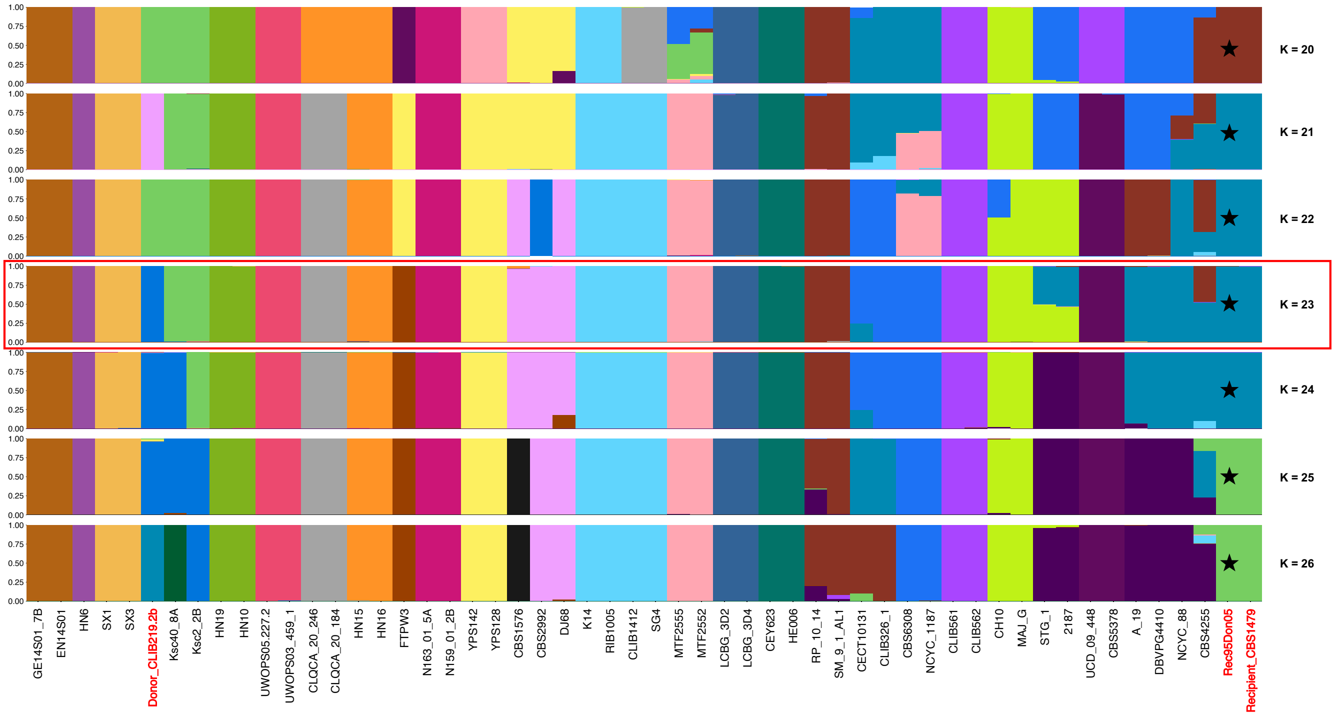

Figure S2: **No effect of 5% contamination on analysis of genetic admixture.** The plot shows the ancestry proportions for each individual arranged in the order seen in Figure S4. These plots show the runs with the highest log-likelihoods for each assumed number of populations (K = 20 - 26), and the run with the most clustering similarity to the phylogenetic analyses (K = 23) is highlighted with a red box. Individual genomes highlighted with red text are the contaminating genome (Donor_CLIB10_2B), and the recipient genome showing the ancestry proportions expected with 0% contamination (Recipient_CBS1479) and an *in silico* mix of recipient with 5% contamination from the contaminant (Rec95Don05, black star) showing the same results as the uncontaminated recipient.

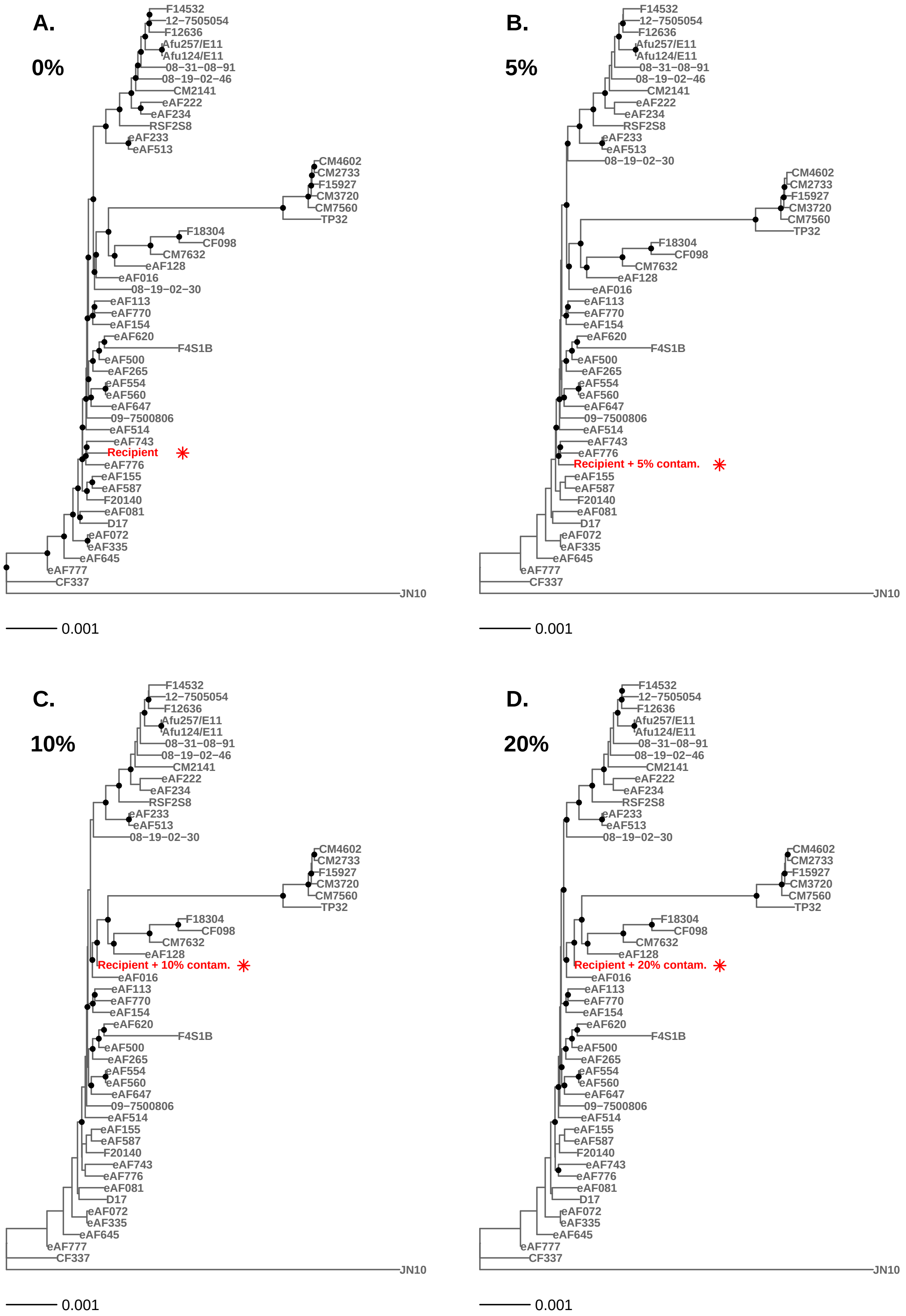

Figure S3: **Change in topology for neighbor joining *A. fumigatus* phylogenetic trees with 10% cross-contamination.** Panel A shows the recipient genome in the absence of cross-contamination; B shows the recipient with 5% contamination; C shows a new topology at 10% contamination and D shows the same new topology at 20%. No contaminant strain was included, and dots at nodes represent bootstrap support of at least 95%.

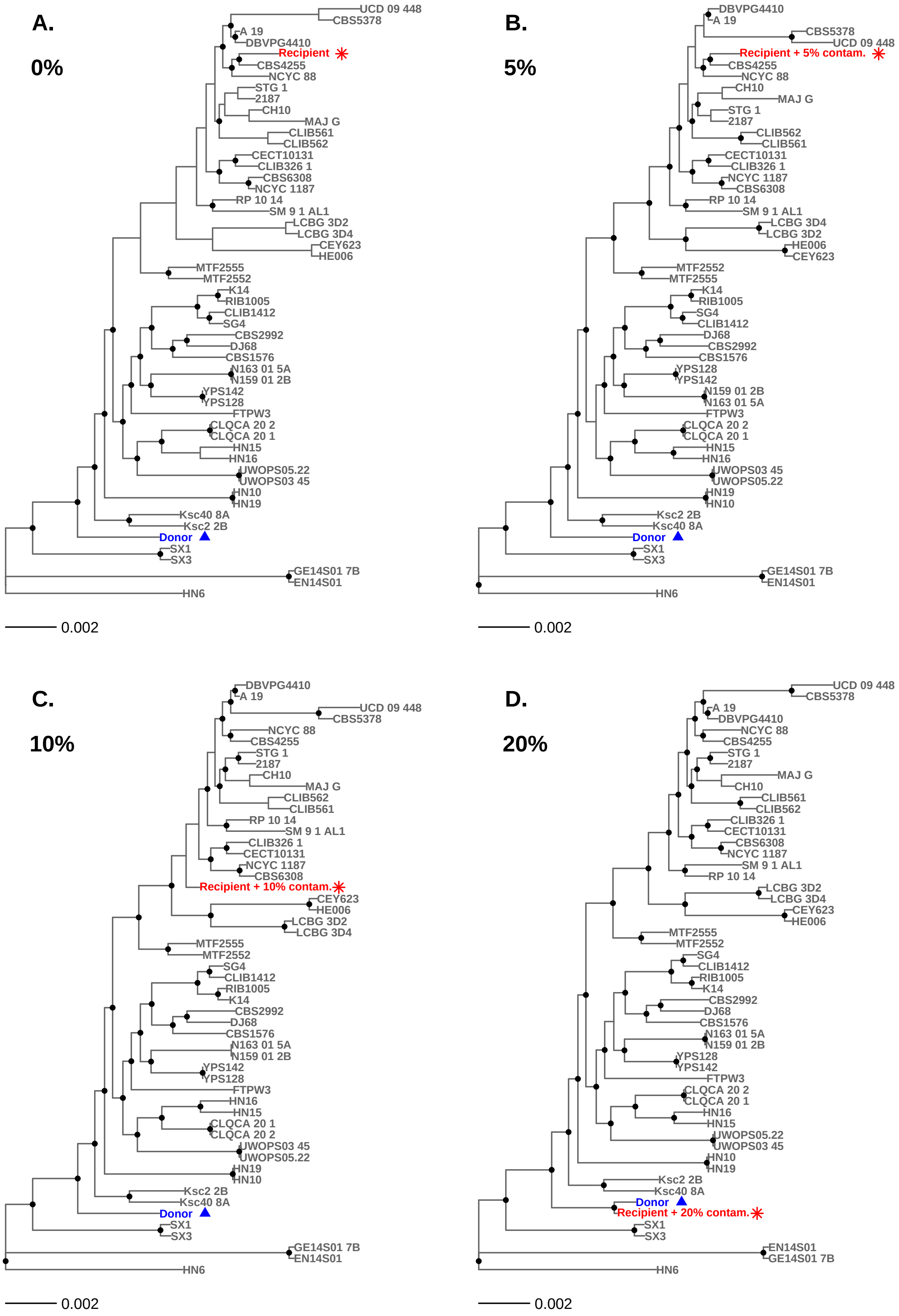

Figure S4: **Change in topology for maximum likelihood *S. cerevisiae* phylogenetic trees with 10% cross-contamination.** Panel A shows contaminant and recipient genomes in the absence of cross-contamination; B shows the recipient with 5%; C shows a new topology at 10%; D shows a more greatly altered topology at 20% contamination. Dots at nodes represent bootstrap support of at least 95%.

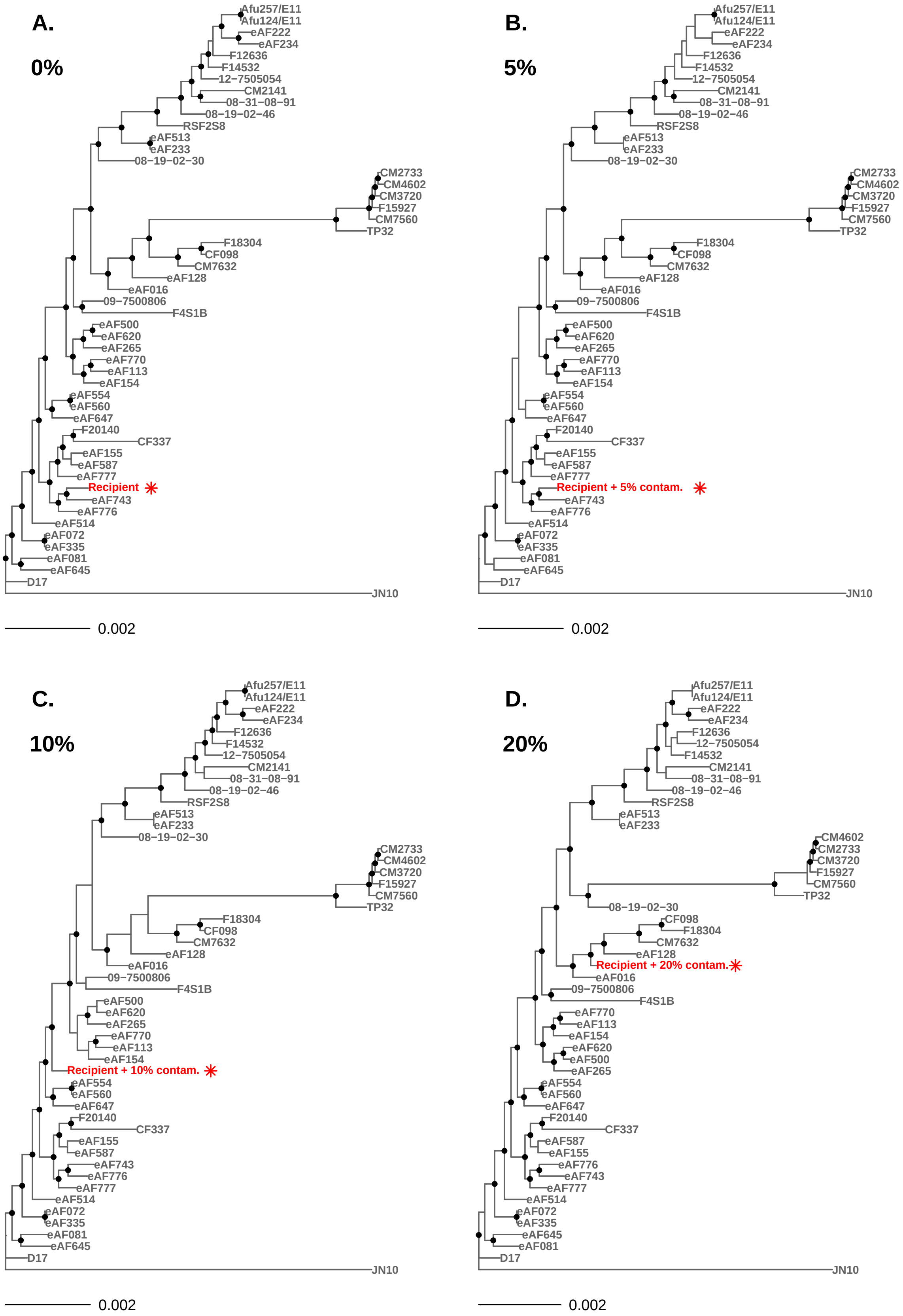

Figure S5: **Change in topology for maximum likelihood *A. fumigatus* phylogenetic trees with 10% cross-contamination.** Panel A shows the recipient genome in the absence of cross-contamination; B shows the recipient with 5%; C shows a new topology at 10%; D shows a more greatly altered topology at 20% contamination. No donor strain was included, and dots at nodes represent bootstrap support of at least 95%.

References

Almeida, P., Barbosa, R., Zalar, P., Imanishi, Y., Shimizu, K., Turchetti, B., Legras, J.L., Serra, M., Dequin, S., Couloux, A., Guy, J., Bensasson, D., Gonçalves, P., and Sampaio, J.P. (2015). "A population genomics insight into the Mediterranean origins of wine yeast domestication." *Molecular Ecology*, **24**(21): 5412–5427.

Barbosa, R., Almeida, P., Safar, S.V.B., Santos, R.O., Morais, P.B., Nielly-Thibault, L., Leducq, J.B., Landry, C.R., Gonçalves, P., Rosa, C.A., and Sampaio, J.P. (2016). "Evidence of Natural Hybridization in Brazilian Wild Lineages of Saccharomyces cerevisiae." *Genome Biology and Evolution*, **8**(2): 317–329.

Bolger, A.M., Lohse, M., and Usadel, B. (2014). "Trimmomatic: a flexible trimmer for Illumina sequence data." *Bioinformatics*, **30**(15): 2114–2120.

Duan, S.F., Han, P.J., Wang, Q.M., Liu, W.Q., Shi, J.Y., Li, K., Zhang, X.L., and Bai, F.Y. (2018). "The origin and adaptive evolution of domesticated populations of yeast from Far East Asia." *Nature Communications*, **9**(1): 2690.

Peter, J., Chiara, M.D., Friedrich, A., Yue, J.X., Pflieger, D., Bergström, A., Sigwalt, A., Barre, B., Freel, K., Llored, A., Cruaud, C., Labadie, K., Aury, J.M., Istace, B., Lebrigand, K., Barbry, P., Engelen, S., Lemainque, A., Wincker, P., Liti, G., *et al.* (2018). "Genome evolution across 1,011 *Saccharomyces cerevisiae* isolates." *Nature*, **556**(7701): 339–344.

Peña, J.J., Scopel, E.F.C., Ward, A.K., and Bensasson, D. (2025). "Footprints of Human Migration in the Population Structure of Wild Baker's Yeast." *Molecular Ecology*, **Early View**(Online): e17669.
